# Cytokine Release Syndrome: A Case Report

**DOI:** 10.7759/cureus.96645

**Published:** 2025-11-12

**Authors:** Inês Filipa Silva, Sofia Gomes Salgueira, Ana Isabel Loureiro, Luisa Nascimento, Teresa Gomes

**Affiliations:** 1 Pulmonology Department, Unidade Local de Saúde de Trás-os-Montes e Alto Douro, Vila Real, PRT

**Keywords:** clinical case report, cytokine release syndrome, diagnosis of rare cases, interleukin-6, interleukin (il)-6, pembrolizumab

## Abstract

Although generally well tolerated, immune checkpoint inhibitors can occasionally lead to severe immune-related adverse events, including cytokine release syndrome (CRS), a condition resulting from excessive immune activation and cytokine secretion, specifically interleukin-6 (IL-6). We report the case of a 71-year-old male with stage IVB lung adenocarcinoma undergoing maintenance therapy with pembrolizumab and pemetrexed, who presented with an acute confusional state and fever unresponsive to antipyretics. An extensive infectious, neurological, and oncological workup was negative. Elevated serum IL-6 levels (270 pg/mL) supported the diagnosis of CRS. The patient was successfully treated with systemic corticosteroids, resulting in the resolution of fever and neurological symptoms. This case underscores the importance of early recognition and prompt management of CRS in patients receiving immune checkpoint inhibitors, highlighting corticosteroid therapy as an effective treatment strategy.

## Introduction

Pembrolizumab is a selective humanized monoclonal antibody that inhibits the programmed cell death-1 (PD-1) receptor on T-cells, thereby enhancing antitumor immune activity [1]. The advent and widespread application of immunotherapy in the treatment of oncological disease have demonstrated significant benefits, particularly in controlling the disease and, consequently, improving survival. Despite their clinical benefits, these agents can lead to immune-related adverse events, ranging from mild to life-threatening.

Among these, cytokine release syndrome (CRS) is a supraphysiological response to immune therapy that activates T cells and other immune effector cells. This systemic reaction is associated with the release of interferon-gamma (IFN-γ) from activated T cells or tumor cells. IFN-γ, in turn, activates macrophages, leading to excessive production of interleukin-6 (IL-6). Diagnosis of this condition is based on clinical features such as the presence of fever, hypotension, hypoxia, and other organ dysfunction, along with increased serum levels of IL-6 [2]. Given the increasing use of immune checkpoint inhibitors, awareness of CRS is crucial for early diagnosis and timely intervention. Here, we present a case of CRS associated with pembrolizumab therapy in a patient with advanced lung adenocarcinoma.

## Case presentation

A 71-year-old male, a former agricultural worker and non-smoker, previously autonomous and cognitively intact, with a family history of a parent with lung cancer, medical history of being overweight, hypertension, dyslipidemia, type 2 diabetes mellitus, and hyperuricemia, was diagnosed in October 2023 with stage IVB left lung adenocarcinoma, demonstrating intermediate programmed death-ligand 1 (PD-L1) expression (25-50%). Given this, he underwent four cycles of chemoimmunotherapy with carboplatin, pemetrexed, and pembrolizumab, followed by maintenance therapy with pemetrexed and pembrolizumab, during which he remained free of immune-related adverse events.

During the third cycle of maintenance therapy, the patient presented to the emergency department with a three-day history of acute confusional state, characterized by disorientation, hallucinations, and aggressiveness, accompanied by fever refractory to antipyretic treatment. On admission, the patient exhibited a Glasgow Coma Scale score of 12. Vital signs were as follows: blood pressure 148/78 mmHg, heart rate 78 breaths per minute (bpm), oxygen saturation 96% on ambient air, and body temperature 39.9 °C. Laboratory evaluation revealed leukocytosis with neutrophilia, mild anemia, and elevated inflammatory markers, with preserved liver and renal function and no evidence of organ failure (Table 1).

**Table 1:** Laboratory parameters at admission in the emergency department Reference intervals are provided for comparison.

Variable	Value	Reference ranges
Hemoglobin	10.60 g/dL	13.00-18.00
Leukocytes	22.21 x 10^3^/uL	4.00-11.00
Neutrophils	93.7 x 10^3^/uL	1.50-8.00
Urea	77 mg/dL	0-50
Creatinine	0.9 mg/dL	0.70-1.20
Lactate Dehydrogenase (LDH)	681 U/L	135-225
Aspartate Aminotransferase (AST)	30 U/L	< 40
Alanine Aminotransferase (ALT)	24 U/L	< 41
Gamma-Glutamyl Transferase (GGT)	178 U/L	10-49
Total Bilirubin	0.4 mg/dL	< 1.2
C-reactive protein (CRP)	12.80 mg/dL	< 0.5
Procalcitonin	0.8 ng/mL	0.5 – Low Risk 0.5 – 1.0 – Moderate Risk > 1.0 – High Risk

Thoracic imaging studies (radiography and computed tomography (CT) revealed a parahilar pulmonary tumor lesion in the left lower lobe, associated with a pleural effusion, both of which were smaller when compared to imaging at diagnosis, with no other new findings. A brain CT excluded an acute cerebrovascular event, hemorrhage, or space-occupying lesion. Empiric broad-spectrum antibiotic therapy for infection in an immunocompromised patient was instituted. The microbiological evaluation, including lumbar puncture, diagnostic thoracentesis, and microbiological cultures, did not reveal any pathogenic organisms (Table 2). 

**Table 2:** Results of microbiological cultures and screenings

Microbiological cultures – Bacteriological, Mycobacterial, and Fungal	
Urine culture	No organisms isolated
Blood cultures	No organisms isolated
Respiratory virus testing	Negative
MRSA and VRE screening	Negative
Cerebrospinal fluid (CSF)	No organisms isolated
Pleural fluid	No organisms isolated

**Figure 1:**
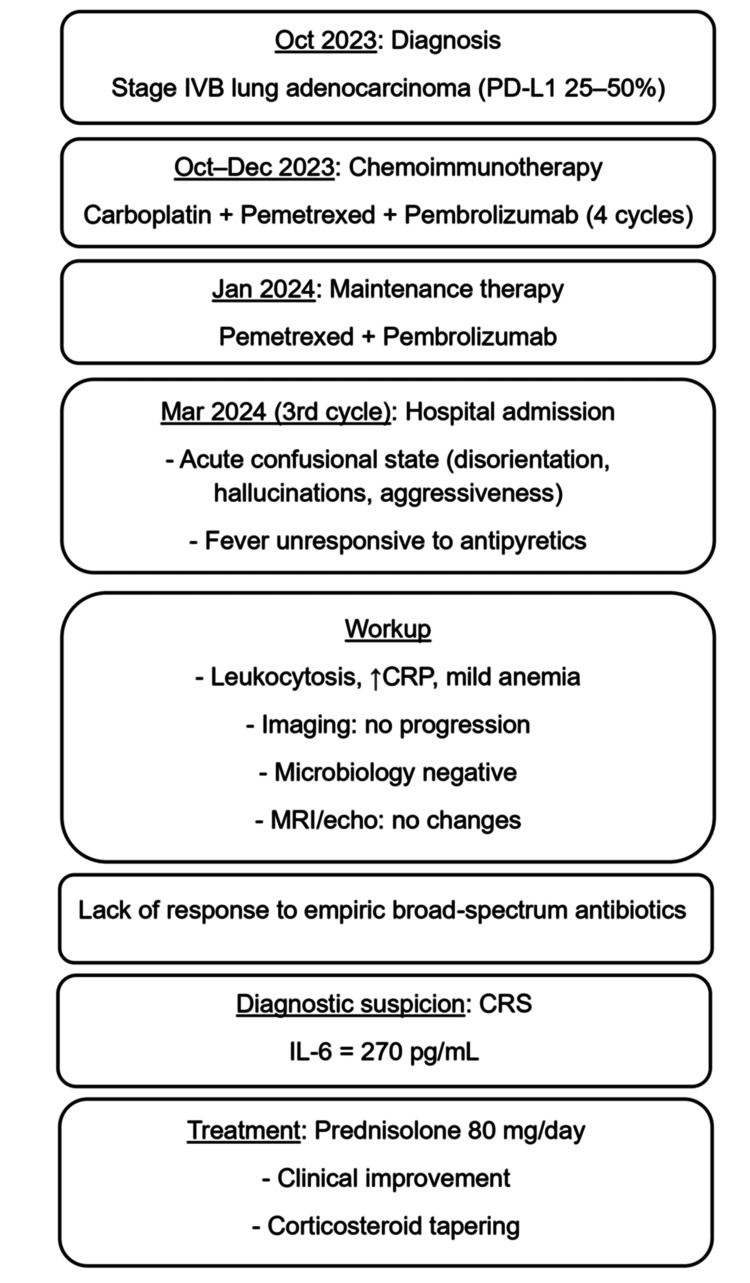
Timeline of clinical events, diagnostic workup, and therapeutic interventions PD-L1: Programmed death-ligand 1; CRP: C-reactive protein; CRS: Cytokine release syndrome.

Transthoracic echocardiogram and brain magnetic resonance imaging were performed to clarify the etiology, which did not reveal any changes. Due to the lack of clinical and analytical response to antibiotic therapy and no evidence of progression of the oncological disease, the diagnosis of CRS was considered. In this context, serum levels of IL-6 = 270 pg/mL (reference range: < 7) confirmed the diagnosis. Thus, weight-adjusted systemic corticosteroid therapy (prednisolone 80 mg/day) was initiated, along with symptomatic supportive care. Consequently, the patient’s temperature normalized, and the acute confusional state resolved. Given the clinical improvement after seven days of corticosteroid therapy without adverse effects, systemic corticosteroid tapering was initiated during hospitalization. A visual timeline of diagnostic and therapeutic events is presented in Figure 1, reinforcing the temporal association between immune checkpoint inhibitor therapy, CRS onset, and the excellent response to corticosteroid therapy.

## Discussion

CRS is an uncommon but potentially severe complication of immune checkpoint inhibitor therapy. It often presents with nonspecific manifestations such as fever, hypotension, hypoxia, and neurological disturbances, which may delay recognition [3]. In the present case, the occurrence of an acute confusional state in conjunction with elevated IL-6 levels strongly suggested CRS, underscoring the importance of early recognition during immunotherapy.

The absence of infectious, neurological, or oncological causes in our patient highlights the diagnostic challenge posed by CRS, particularly when routine investigations are inconclusive. Biomarker correlation, especially the marked elevation of IL-6, supported the diagnosis in this case. Our findings align with previous reports indicating that CRS may develop even in patients without prior immune-related adverse events, emphasizing the need for vigilance across all patient populations [2]. 

The median time between immune checkpoint inhibitor initiation and development of CRS was four weeks, although a great heterogeneity in timelines is possible due to the therapy used or individual characteristics [3]. Our case consisted of a five-month period between the two events, and although far greater than mentioned, longer periods have been described in literature, such as two years after pembrolizumab initiation [2]. 

The management of CRS involves discontinuation of the immune checkpoint inhibitor, administration of systemic corticosteroids, and supportive care. In patients with severe CRS, the administration of tocilizumab, combined with corticosteroids, has been associated with a more rapid and complete resolution of symptoms [4]. The case reported by Nakashima et al. involved a patient with an even more delayed diagnosis, which translated into greater severity with cardiovascular and hematologic dysfunction; however, a complete clinical response to only corticosteroid and supportive care was achieved [2].

Resolution of the acute neurological episode after corticosteroid therapy, with normalization of consciousness and a return to baseline cognitive and functional status, was sustained after follow-up in an outpatient setting. This favorable outcome underscores the potential for full recovery when cytokine release syndrome is promptly recognized and appropriately managed, highlighting the importance of early intervention and close monitoring in patients receiving immune checkpoint inhibitors.

This case illustrates the importance of early recognition and timely treatment of CRS in patients receiving pembrolizumab. Although rare, this condition can be life-threatening and requires a high degree of clinical suspicion because of its ambiguous presentation [5]. Despite its single-case nature, this report reinforces the need for clinical awareness of this rare but serious immune-related adverse event, as prompt intervention can significantly improve outcomes.

## Conclusions

In conclusion, CRS represents a rare but potentially life-threatening adverse effect of immune checkpoint inhibitors, requiring prompt recognition and timely intervention. This case highlights the importance of early identification of elevated IL-6 levels, together with the thorough exclusion of infectious and oncological etiologies. These steps are critical for accurate diagnosis and underscore the diagnostic challenge often posed by this condition. While corticosteroid therapy was effective in this patient, other therapeutic strategies, such as tocilizumab, may also play a role in the management of severe cases. Furthermore, this report underscores the clinical significance of CRS in the context of immune checkpoint inhibition, a phenomenon that has been only sparsely documented to date. In light of the growing prevalence of anti-PD-L1 therapies, heightened clinical vigilance is imperative to ensure timely recognition and management of this potentially serious treatment-related complication.
